# Apollo2Go: a web service adapter for the Apollo genome viewer to enable distributed genome annotation

**DOI:** 10.1186/1471-2105-8-320

**Published:** 2007-08-30

**Authors:** Kathrin Klee, Rebecca Ernst, Manuel Spannagl, Klaus FX Mayer

**Affiliations:** 1 MIPS/Institute for Bioinformatics, GSF Research Center for Environment and Health, Ingolstädter Landstrasse 1; 85764 Neuherberg, Germany

## Abstract

**Background:**

Apollo, a genome annotation viewer and editor, has become a widely used genome annotation and visualization tool for distributed genome annotation projects. When using Apollo for annotation, database updates are carried out by uploading intermediate annotation files into the respective database. This non-direct database upload is laborious and evokes problems of data synchronicity.

**Results:**

To overcome these limitations we extended the Apollo data adapter with a generic, configurable web service client that is able to retrieve annotation data in a GAME-XML-formatted string and pass it on to Apollo's internal input routine.

**Conclusion:**

This Apollo web service adapter, Apollo2Go, simplifies the data exchange in distributed projects and aims to render the annotation process more comfortable.

The Apollo2Go software is freely available from .

## Background

The number of finished bacterial genomes and higher eukaryotic genomes is dramatically increasing. Consequently there is a pressing need for accurate annotation of the respective genomes and, even more important, for the maintenance of genome annotation. With the increasing complexity, and the large number of the genomes on one hand and the only limited availability of appropriate and sufficient resources for annotation and curation on the other there is a strong demand for solutions and tools to assist in community-centric, decentralized and distributed genome annotation for maintenance of high quality genome annotation. In addition, genome sequencing projects are often set up as distributed, collaborative projects with contributions from multiple research groups. A necessity for such approaches is the implementation of common data exchange formats and an annotation system that enables remote users to edit and curate database entries. Such a system should be user friendly, flexible and easy to implement.

Several approaches have been proposed to address these problems. A popular and widely applied approach is to employ experts for an initial genome annotation within genome annotation jamborees. Examples comprise the annotation of the rice genome, the *Drosophila *genome and the Human Genome Project Consortium's Analysis Group (HGPCAG) [1-3]. These approaches aim to overcome the problem of collecting distributed data by bringing together scientists, harvest their joint expertise and integrate the annotations into the respective database system.

A different approach is to distribute not only the annotation efforts but also the data management by using a distributed annotation system (DAS) browser that integrates and displays annotations from various sources [4]. DAS annotations are available for numerous genomes and provide a powerful means to integrate different and decentralized annotations.

However, to our knowledge currently there is no DAS client software available that provides functionality to display, integrate, annotate and re-write data in a standardized way.

Finally, a third approach is frequently used to integrate contributions from several groups. In this approach, a web accessible annotation interface is made available along with visualization tools and complementary analysis data. With the genome browser visualization interface along with complementary analysis data contributors are enabled to view and annotate database entries and finally to submit them back into the reference database. In our work we are following this approach by adopting and advance the Apollo genome annotation and visualization tool.

For annotation, Apollo is established as a widely used genome annotation and visualization tool. Beside local usage, Apollo has been used in distributed genome annotation projects [5]. After completion of the annotation process a database update is carried out by uploading the annotation files. Albeit robust the non-direct database upload via Apollo's intermediate GAME-XML formatted files is a tedious and laborious task and evokes problems of data synchronicity. To overcome these limitations we developed a web service adapter (Apollo2Go) for the Apollo genome annotation tool. Web services recently became widely used tools and provide important advantages, such as platform interoperability, only limited firewall problems, open standards and, importantly, wide acceptance in the scientific community.

The Apollo2Go adapter is a client program that enables the connection to specified web services that serve and accept the annotation data. The adapter combines the advantages of a well established and widely used annotation software with the convenience of directly accessing and writing data back into the database. Thereby the intermediate processing of GAME-XML files is omitted.

## Implementation

**Figure 1:**
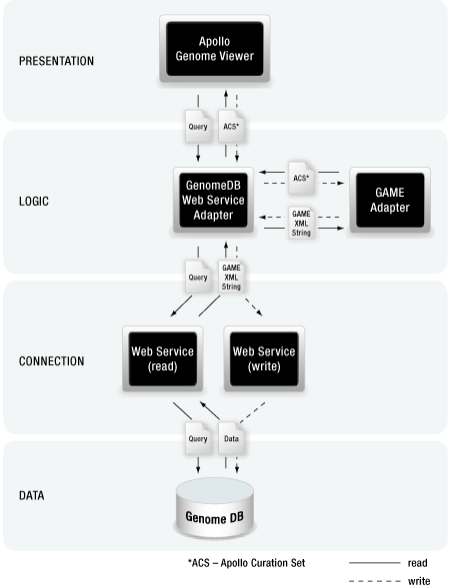
**Apollo web service adapter overview**. **To load data **into Apollo the Apollo GUI calls the web service adapter GUI, which calls the web service adapter. The web service adapter invokes the web service client that communicates with the web service. The web service is deployed on an in house server that directly accesses the database. The database returns the information of interest to the web service and the web service returns it to the web service adapter. The web service returns the information as a GameXML formatted String and the GAME-XML adapter transforms the information into Apollo readable format. Subsequent the information can be loaded from the web service adapter into the Apollo main window. **To write data **back into the database or in a flat file the web service adapter receives the information as CurationSet from Apollo. This CurationSet will be send to the GAME-XML adapter to retrieve a GameXML formatted string, which can be transferred to the web service. The web service directs the information into the database or in a dedicated flat file.

To implement Apollo2Go's web service functionality we added several adapter classes to the Apollo code. We created two GUI interfaces to communicate web service input and output parameters as well as two adapter classes, each concerning the respective web service client for reading and writing data from and to an individual data source. The web service adapter classes that contain the clients were connected to the existing GameXML adapter that allows data to be easily converted into an Apollo-compatible form (see also Figure 1). The web service adapter GUIs can be addressed via the data adapter chooser from Apollo's input and output/save forms.

## Display of annotation data from remote resources

To use Apollo's web service capability, i.e. to be able to display annotation data of selected regions or complete contigs or chromosomes from remote resources, the data provider has to make its data available in Apollo compatible GAME-XML format via a web service. Beside a particular database that hosts annotation data also specified directories that contain the required GAME-XML files can serve as data sources for the web service. Once a web service is set-up, annotations can be accessed using the Apollo2Go web service from any remote computer.

In addition to the Apollo2Go web service-enabled Apollo, the remote user only has to fill in a configuration file which contains the web service dependent variables.

## Writing annotation data to remote resources

After remote display and data edits in Apollo, modified annotations can be inserted into the reference database. Typically, annotation data are stored in a dedicated database that, within the framework of a distributed annotation project, will often be embodied by a non-local resource.

Since there is a variety of different database schemas and concepts, direct insertion of remote, Apollo-curated annotation is impossible. Therefore, each database provider has to make a web service available that is capable to insert annotation data in GAME-XML format into the respective database. All logic that is associated with database insertion like consistency checks, user authorization, as well as insertion itself takes place within the web service.

Thus, similar to the concept of displaying remote data, the configurable Apollo2Go web service client has been integrated into Apollo's 'save' functionality. The authorized user solely has to complete a configuration file to write data back into the specified database. Due to the uncoupling of database schema and display no restrictions with respect to the database schema are apparent for the enhanced Apollo version. An overview on the web service enabled Apollo version and the interplay with the components involved is depicted in Figure 1.

## Results

### Use case: Implementation and Application of Apollo web services for MIPS' PlantsDB

We developed a web service that receives annotation data from the plant databases developed and hosted by our group (MIPS PlantsDB) [6,7]. PlantsDB hosts multiple databases for different genomes and the web service receives data for the given genome database, the respective contig name and the selected sequence range. The Apollo2Go built-in client invokes this web service with arguments derived from the adapter's graphical user interface. The client retrieves the result as a GAME-XML-formatted string and passes it to the Apollo input methods. For data not provided by a database system, the web service also enables to receive data provided as flat files.

After retrieval, the data can be subjected to curation and annotation using Apollo's annotation and visualization interface. In our implementation before write-back into the database all data are tested for redundancy, versioned and inserted into the database. 'Old' data will not be overwritten but are set to a non-visible mode and only more recent and up-to-date annotations are displayed via the public interface. However this functionality has to be provided by the individual web service implementation.

It should be emphasized that usability of the Apollo2Go web service functionality is not restricted to local or in-house use but has successfully been used for non-local annotation of PlantsDB database entries.

## Conclusion

The Apollo2Go web service adapter is a powerful extension to the Apollo genome editor. It is ideally suited for manual genome annotation and curation in distributed environments. By combining the widely applied Apollo genome editor with web service functionality, the data exchange is more efficient and the annotation process is more comfortable. The implementation has been made generic. Therefore the method is not restricted to a specific genome data provider or database schema but can be used for different settings and environments. In addition, extended functionality that allows for complex features like security checks before insertion of updated data into the database or data repository may be added to the web service.

Within the annotation process curators are enabled to connect to a remote database, collect the sequences of interest and to display and edit within Apollo. After finishing of the annotation process, the modified data can be directly inserted into the remote database. The necessary read and write access to the database can be secured via password controlled access. Any center hosting genome and annotation data can enable access to the respective data by hosting web services to either serve the data that allows other groups to display it in their Apollo installation or to accept annotated data from approved curators.

## Availability and requirements

Project name: Apollo2Go

Project home page: 

Operating systems: Platform independent (Apollo is now set up to run with JDK1.5 and it runs on Windows, Unix, Linux and Mac platforms).

Programming language: Java; Other requirements: Java 1.5

License: The Apollo JAVA code is Open Source; Apollo is distributed under the terms of the Artistic License  and the Apollo source code is available at SourceForge  as part of the GMOD project . We plan to include the Apollo web service adapter package as soon as possible at this place.

Any restrictions to use by non-academics: see terms of the Artistic License .

The Apollo2Go software package is available via anonymous FTP from .

The zipped TAR file contains Apollo2Go, the latest executable Apollo genome annotation curation tool (Version 1.6.5, last updated June 29, 2006) extended with the build-in web service adapter (including the example to connect to PlantsDB at MIPS). The Apollo2Go software does not need any additional local installation of Apollo. Memory requirements are unchanged to Apollo's memory requirements and largely depend on the genomic region selected for annotation. At least 164 Mb of RAM, preferably more, are recommended.

## Authors' contributions

KK carried out the programming work, designed the web service and integrated the web service into Apollo. RE conceived the work and participated in the web service design and draft manuscript. MS contributed the database connectivity of Apollo2Go and contributed to the draft manuscript. KFXM conceived and supervised the work and wrote the manuscript. All authors read and approved the final manuscript.
